# Supplementary material for: British Gynaecological Cancer Society Recommendations for Evidence Based, Population Data Derived Quality Performance Indicators for Ovarian Cancer
Source: Cancers (Basel). 2023 Jan 4;15(2):337. doi: 10.3390/cancers15020337 (PMC9856668; doi:10.3390/cancers15020337)
Supplement: Supplementary file 1 [file cancers-15-00337-s001.zip › BGCS QPI Box1.pdf]

1	One-stop diagnostic clinics where patients can receive expert ultrasound/ CT imaging/ image-guided biopsies to confirm malignancy and stratify to the appropriate pathways<br><br>Pathways for women at low risk for malignancy to be managed in a standardised manner
2	Facilitation of outpatient interventions to improve diagnostic pathways in patients presenting as emergencies: ascites pathway; image-guided biopsies; standards for ascites molecular testing; acute oncology assessments
3	Interventions for standardisation of decision making or treatment: innovative frailty assessments/ standardisation of radiology reporting)
4	Improving culture around surgical expertise: standardization of operative notes; defining supportive care for high tumour burden patients undergoing extensive surgery; surgical mentorship schemes
5	More complete and granular data to inform future iterations of audit (e.g. comparing local data from coding against national data, developing automated pipelines for local data)
6	Addressing equity of access for ethnic minority patients and those with higher levels of socially deprivation to specialised care and genetic testing
7	Implementation of genetic testing, including tumour testing in referring hospitals (secondary care)

Box S1 - Best practice recommendations to optimise clinical care in advanced ovarian cancer
