# Supplementary material for: British Gynaecological Cancer Society Recommendations for Evidence Based, Population Data Derived Quality Performance Indicators for Ovarian Cancer
Source: Cancers (Basel). 2023 Jan 4;15(2):337. doi: 10.3390/cancers15020337 (PMC9856668; doi:10.3390/cancers15020337)
Supplement: Supplementary file 1 [file cancers-15-00337-s001.zip › BGCS QPI Box2.pdf]

**At cancer service provider level**	Gynaecological MDT leads should examine their individual centre/unit data on dedicated registry platforms (ie.CancerStats2 platform) and compare these against the proposed Quality Performance Indicators QPI's. Granular local data may shed more insight into actions that are needed at each site.<br><br>A subsequent status report and actions plan to Cancer boards/ Cancer Alliances as a response to CancerStats2 data is encouraged.<br><br>Hospital/centre/alliance support is a prerequisite to perform this task as a priority.
**Action for Cancer Alliances**	Each Cancer Alliance is encouraged to review action plans by Trusts providing cancer services in response to findings from the ovarian cancer audit.<br><br>An example would be the implementation of supra-regional MDTs and an Operational Delivery network to harmonise best practice building on the infrastructure embedded through digital pathology and genomics initiatives. This will enable discussion of selected patients from across entire regions to drive standardisation of decision-making.
**Actions for Integrated Care Systems**	Integrated Care Systems must address the issues around delay in diagnostic pathways with targeted interventions in Clinical Commissioning Groups (CCGs) where data show a greater proportion of patients diagnosed at advanced and unstaged disease.<br><br>The BGCS notes ongoing efforts with the NHS Long Term Plan such as rapid diagnostic clinics, diagnostic centres and faster diagnosis standards that will help improve diagnostic pathways for ovarian cancer.

Box S2 - Actions needed in response to the UK Ovarian cancer audit; findings from Cancer services providers, Cancer Alliances and Integrated care systems
