# Supplementary material for: British Gynaecological Cancer Society Recommendations for Evidence Based, Population Data Derived Quality Performance Indicators for Ovarian Cancer
Source: Cancers (Basel). 2023 Jan 4;15(2):337. doi: 10.3390/cancers15020337 (PMC9856668; doi:10.3390/cancers15020337)
Supplement: Supplementary file 1 [file cancers-15-00337-s001.zip › BGCS QPI Box3.pdf]

1	Definition of algorithms for patient-stratification to appropriate diagnostic and treatment pathway
2	Definition and validation of gynaecological oncology-specific frailty scores and complication scores
3	Linkage of socioeconomic data with genetic testing data and exploration of barriers to the broad implementation of genetic testing
4	Utilise the audit infrastructure to establish linked networks of tissue banks that can use routinely-collected data and outcomes for translational research as well as with prospectively assessed comprehensive clinical data
5	Define subgroup of patients who will not benefit from standard treatment approaches and need to be directed towards alternative treatment pathways at diagnosis
6	Health systems research to investigate effectiveness of supra-regional networks for ovarian cancer

Box S3 - Priorities and key aspects for research
