# Supplementary material for: British Gynaecological Cancer Society Recommendations for Evidence Based, Population Data Derived Quality Performance Indicators for Ovarian Cancer
Source: Cancers (Basel). 2023 Jan 4;15(2):337. doi: 10.3390/cancers15020337 (PMC9856668; doi:10.3390/cancers15020337)

Figure S1. Net survival rates of patients with ovarian, fallopian tube and primary peritoneal carcinomas -excluding borderline histologies (all stages) at 1- and 5-years post diagnosis, by Cancer Alliance (diagnoses 2013 to 2017)

From the National Ovarian Cancer Audit Feasibility Report [13]

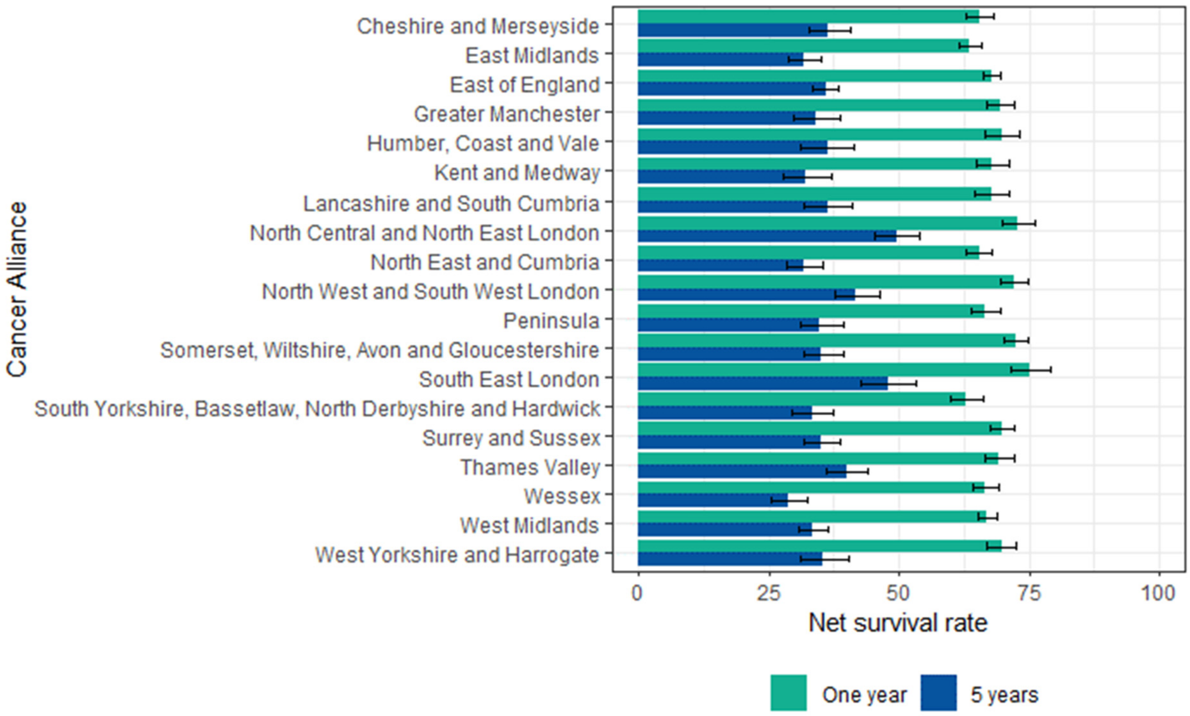

Supplement: Supplementary file 1 [file cancers-15-00337-s001.zip › BGCS QPI Figure 1.pdf]
