# Supplementary material for: British Gynaecological Cancer Society Recommendations for Evidence Based, Population Data Derived Quality Performance Indicators for Ovarian Cancer
Source: Cancers (Basel). 2023 Jan 4;15(2):337. doi: 10.3390/cancers15020337 (PMC9856668; doi:10.3390/cancers15020337)
Supplement: Supplementary file 1 [file cancers-15-00337-s001.zip › File S1. OVARIAN CA OP REPORT - Oct 2020[40].pdf]

ROYAL MARSDEN HOSPITAL OVARIAN CANCER OPERATIVE REPORT

1. PATIENT INFORMATION

Hospital no.		Date of birth		Date of Surgery		WHO PS	Karnof PS	ASA grade
BMI	Pre-Op:	Hb	Albumin	Ca-125	CEA	Ca-19,9	Ca-15,3	others

2. SURGERY DATA

**1st Surgeon:**		**2nd Surgeon:**		**Knife to skin (HH:mm):**		**Type of tumour:**	Primary	Recurrent
**Aim of surgery (circle):**	Diagnostic	Staging	Primary CRS	Interval CRS	Secondary CRS	Tertiary CRS	Re-staging	Palliative	Emergency

**Suspected or Confirmed Diagnosis:**				**FIGO Stage (Table 1):**		
**Suspected Stage IV?** Yes No	Pleural effusion	Y N	Positive cytology	Y N n/a	Pleural / lung parenchyma	Y N
(before chemotherapy)	Liver parenchyma	Y N	Spleen parenchyma	Y N	Other (specify):	
	Extra-abdominal lymph nodes?	Y N		Location:		

3. SURGICAL APPROACH AND FINDINGS

**Previous gynaecological resection (circle):**	Yes	No		**If previous surgery, list organs resected:**			
**Approach (circle):**	Open	Laparoscopic	Robotic	**Volume of Ascites (circle):**	0 mL	1 – 500 mL	>500 mL
**Intraoperative surgical spillage:**	Yes	No		**Capsule rupture prior to surgery:**	Yes	No	
**Frozen Section (circle):**	Yes	No		**Frozen Section Diagnosis:**			

Y=yes / N=no / U=unknown

TUMOUR INVOLVEMENT

Right ovary		Vagina		Pelvic sidewall		Omentum		Para-aortic LNs		Lesser omentum		Coeliac LNs	
Right tube		Uterus		Pelvic LNs		Large bowel		R diaphragm		Stomach		Abdominal wall	
Left ovary		Bladder / ureter		R paracol gutter		Appendix		L diaphragm		Pancreas		Skin	
Left tube		Sigmoid / rectum		L paracol gutter		Sm bowel mesen.		Liver surface		Spleen		Peri-cardiophrenic LNs	
Pouch of Douglas		Recto-vag septum		Small bowel		Lg bowel mesen.		Liver parenchyma		Hepatic hilum LNs		Inguinal LNs	

Peritoneal Cancer Index Scoring

Score each section 0-3 pre and postoperatively:

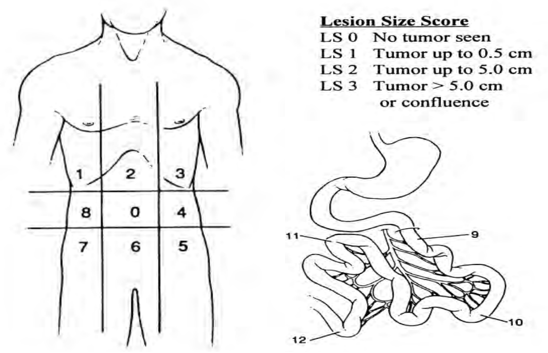

PERITONEAL CANCER INDEX

	PRE	POST
0 Central		
1 Right upper		
2 Epigastrium		
3 Left upper		
4 Left flank		
5 Left lower		
6 Pelvis		
7 Right lower		
8 Right flank		
9 Upper jejunum		
10 Lower jejunum		
11 Upper ileum		
12 Lower ileum		
**PCI**		

Anatomical Landmarks for Assessing Residual Retroperitoneal Disease

Tick all that apply:

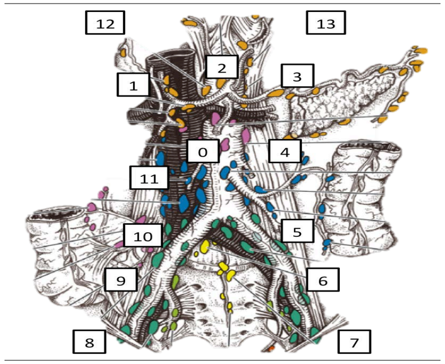

RETROPERITONEAL DISEASE

	+	R+	R0
0 Interaortocava/preaort.			
1 Porta Hepatis			
2 Celiac Axis			
3 Suprarenal/Splenic			
4 Left aortic			
5 Left common iliac			
6 Left ext iliac			
7 Left inguinal			
8 Right inguinal			
9 Right ext iliac			
10 Right common iliac			
11 Pre-Paracava			
12 Right cardio phrenic			
13 Left cardio phrenic			

+: Suspicious or Positive
R+: Residual disease
R0: No residual disease

4. SURGICAL PROCEDURES

Tick all that apply:

☐ Hysterectomy	☐ Pelvic lymphadenectomy	☐ Resection of lesser omentum	☐ Liver capsule resection
☐ Unilateral salpingo-oophorectomy	☐ Peritonectomy of paracolic gutters	☐ Partial gastrectomy	☐ Atypical liver resection (explain):
☐ Bilateral salpingo-oophorectomy	☐ Para-aortic lymphadenectomy	☐ Coeliac lymphadenectomy	☐ Partial hepatectomy
☐ Small bowel mesentery	☐ Small bowel resection	☐ Hepatic hilum lymphadenectomy	☐ Cholecystectomy
☐ Ureteral resection	☐ Large bowel resection	☐ Diaphragmatic stripping	☐ Morrison's Pouch peritonectomy
☐ Colorectal resection	☐ Appendicectomy	☐ Diaphragmatic resection	☐ Inguinal lymphadenectomy
☐ Partial cystectomy	☐ Infracolic omentectomy	☐ Splenectomy	☐ Peri-cardiophrenic lymphadenectomy
☐ Pelvic peritonectomy	☐ Radical omentectomy	☐ Partial pancreatectomy	☐ PlasmaJet

**Number Anastomoses:** 0 1 2 3 4	**Residual small bowel (cm):**	**Stoma Formation:** yes no	**Stoma Type:** Definitive Temporary N/A

Other procedures (circle): IP-Port-a-cath IV-Port-a-cath Abdominal wall resection Mesh placement VATS HIPEC None
**Further description of surgical procedures (eg: type of anastomoses, resection, stoma, indication for PlasmaJet):**

5. RESIDUAL DISEASE

**Intra-abdominal (circle):**	No macroscopic	0.1-0.59cm	0.6-1.0cm	>1cm		
**Extra-abdominal (circle):**	No macroscopic	0.1-0.59cm	0.6-1.0cm	>1cm		
**Location (describe):**						
**Reason(s) for no complete debulking:** ***see below**	1	2	3	4	5	6
**Consultant second opinion sought? (circle):**	YES	NO				
**CC-score (completeness of cytoreduction):**	CC-0= no peritoneal nodule was seen<br>CC-1= nodules persisting after cytoreduction <2.5mm<br>CC-2= nodules between 2.5mm and 2.5cm<br>CC-3= nodules >2.5cm or a confluence of unresectable tumour nodule at any site within the abdomen or pelvis					
**GOG-score (max diameter of residual deposits):**	1 = no macroscopic disease	2 = 0.1-1cm	3 = 1-2cm	4 = >2cm		

6. END OF OPERATION

**Surgery finish time (HH:mm):**	**Estimated blood loss (mL):**	**Antibiotics:**	**Blood products transfused:**

**Intraoperative complications:**						
**Patient admitted to ITU with (circle)**	Inotropes	NGT tube	Epidural Catheter	Endotracheal tube	Chest tube	Drain(s):
**Free text comments:**						

* 1= diffuse deep infiltration of the root of small bowel mesentery, 2= diffuse carcinomatosis of the small bowel that requires resection leading to short bowel syndrome (<1.5m), 3= diffuse involvement / infiltration of stomach/duodenum, 4= diffuse involvement / deep infiltration of head or middle part of pancreas, 5= involvement of truncus coeliacus, hepatic arteries or left gastric artery, 6= non-resectable enlarged lymph nodes

Table S1
2014 FIGO ovarian, fallopian tube, and peritoneal cancer staging system and corresponding TNM.

I	Tumor confined to ovaries or fallopian tube(s)	T1
IA	Tumor limited to one ovary (capsule intact) or fallopian tube, No tumor on ovarian or fallopian tube surface No malignant cells in the ascites or peritoneal washings	T1a
IB	Tumor limited to both ovaries (capsules intact) or fallopian tubes<br>No tumor on ovarian or fallopian tube surface<br>No malignant cells in the ascites or peritoneal washings	T1b
IC	Tumor limited to one or both ovaries or fallopian tubes, with any of the following:<br>IC1 Surgical spill intraoperatively<br>IC2 Capsule ruptured before surgery or tumor on ovarian or fallopian tube surface<br>IC3 Malignant cells present in the ascites or peritoneal washings	T1c
II	Tumor involves one or both ovaries or fallopian tubes with pelvic extension (below pelvic brim) or peritoneal cancer (Tp)	T2
IIA	Extension and/or implants on the uterus and/or fallopian tubes/and/or ovaries	T2a
IIB	Extension to other pelvic intraperitoneal tissues	T2b
III	Tumor involves one or both ovaries, or fallopian tubes, or primary peritoneal cancer, with cytologically or histologically confirmed spread to the peritoneum outside the pelvis and/or metastasis to the retroperitoneal lymph nodes	T3
IIIA	Metastasis to the retroperitoneal lymph nodes with or without microscopic peritoneal involvement beyond the pelvis	T1,T2,T3aN1
IIIA1	Positive retroperitoneal lymph nodes only (cytologically or histologically proven)	
IIIA1(i)	Metastasis $\leq$ 10 mm in greatest dimension (note this is tumor dimension and not lymph node dimension)	T3a/T3aN1
IIIA1(ii)	Metastasis > 10 mm in greatest dimension	
IIIA 2	Microscopic extrapelvic (above the pelvic brim) peritoneal involvement with or without positive retroperitoneal lymph nodes	T3a/T3aN1
IIIB	Macroscopic peritoneal metastases beyond the pelvic brim $\leq$ 2 cm in greatest dimension, with or without metastasis to the retroperitoneal lymph nodes	T3b/T3bN1
III C	Macroscopic peritoneal metastases beyond the pelvic brim > 2 cm in greatest dimension, with or without metastases to the retroperitoneal nodes (Note 1)	T3c/T3cN1
IV	Distant metastasis excluding peritoneal metastases	
	Stage IV A: Pleural effusion with positive cytology	Any T, Any N,
	Stage IV B: Metastases to extra-abdominal organs (including inguinal lymph nodes and lymph nodes outside of abdominal cavity) (Note 2)	M1
	(Note 1: includes extension of tumor to capsule of liver and spleen without parenchymal involvement of either organ)	T3c/T3cN1)
	(Note 2: Parenchymal metastases are Stage IV B)	

Notes:
1. Includes extension of tumor to capsule of liver and spleen without parenchymal involvement of either organ.
2. Parenchymal metastases are Stage IV B.
